# Current applications and future perspective of CRISPR/Cas9 gene editing in cancer

**DOI:** 10.1186/s12943-022-01518-8

**Published:** 2022-02-21

**Authors:** Si-Wei Wang, Chao Gao, Yi-Min Zheng, Li Yi, Jia-Cheng Lu, Xiao-Yong Huang, Jia-Bin Cai, Peng-Fei Zhang, Yue-Hong Cui, Ai-Wu Ke

**Affiliations:** 1 Key Laboratory of Carcinogenesis and Cancer Invasion, Ministry of Education, Liver Cancer Institute, Zhongshan Hospital, Fudan University, 180 Fenglin Road, Shanghai, 200032 People’s Republic of China; 2 Institute of Biomedical Sciences, Fudan University, 138 Medical School Road, Shanghai, 200032 People’s Republic of China; 3 Department of Oncology, Zhongshan Hospital, Fudan University, 180 Fenglin Road, Shanghai, 200032 People’s Republic of China

**Keywords:** CRISPR/Cas9, Gene editing, CRISPR screen, Gene delivery, Immunotherapy

## Abstract

Clustered regularly interspaced short palindromic repeats (CRISPR) system provides adaptive immunity against plasmids and phages in prokaryotes. This system inspires the development of a powerful genome engineering tool, the CRISPR/CRISPR-associated nuclease 9 (CRISPR/Cas9) genome editing system. Due to its high efficiency and precision, the CRISPR/Cas9 technique has been employed to explore the functions of cancer-related genes, establish tumor-bearing animal models and probe drug targets, vastly increasing our understanding of cancer genomics. Here, we review current status of CRISPR/Cas9 gene editing technology in oncological research. We first explain the basic principles of CRISPR/Cas9 gene editing and introduce several new CRISPR-based gene editing modes. We next detail the rapid progress of CRISPR screening in revealing tumorigenesis, metastasis, and drug resistance mechanisms. In addition, we introduce CRISPR/Cas9 system delivery vectors and finally demonstrate the potential of CRISPR/Cas9 engineering to enhance the effect of adoptive T cell therapy (ACT) and reduce adverse reactions.

## Introduction

There are many viruses in the environment that threaten the survival of prokaryotes [1]. As a defense mechanism, prokaryotes developed an adaptive immune system called clustered regulatory interspaced short palindromic repeats (CRISPR) [2]. A CRISPR locus consists of spacers derived from bacteriophages and other extrachromosomal elements, separated by short repeated sequences encoding small non-messenger RNA. These spacers prevent infection from their originating viral strains, and they are adaptive—bacteria integrate a new spacer from the phage genome after viral attack, and elimination or addition of specific spacers changes phage resistance of bacteria [3–6]. In addition, there are four CRISPR-associated (*cas*) genes adjacent to the CRISPR locus [7, 8].

**Fig. 1:**
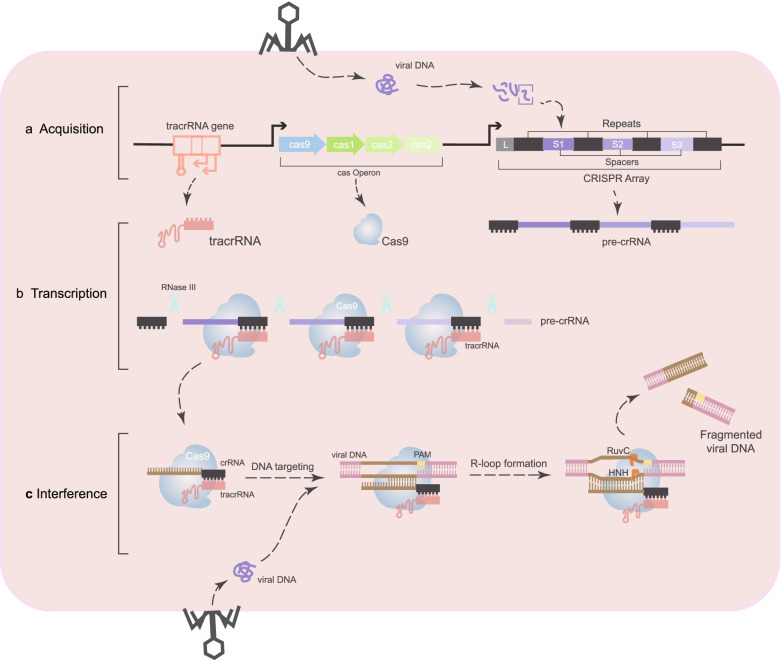
Mechanism of type II CRISPR/Cas9 system. **a** During acquisition, after being infected by the phage, the DNA sequence from the invader is integrated into the host CRIPSPR locus as a spacer and separated by repetitive sequences. **b** During the transcription stage, pre-crRNA is transcribed, and then pre-crRNA is cleaved to produce mature crRNA. Each crRNA is composed of a repetitive sequence and a spacer sequence against the invader. **c** In the interference phase, the Cas protein directly cleaves the exogenous nucleic acid at a site complementary to the sequence of the crRNA spacer

CRISPR/Cas-mediated adaptive immunity occurs over three steps (Fig. 1). First, prokaryotes acquire cellular memory of invading viruses or plasmids [9]. After infection, a DNA sequence from the invader integrates into the host CRISPR locus as spacer arrays flanked by repetitive sequences [6], providing specific phage resistance based on the sequence. Second, RNA polymerase produces RNA from spacer regions of the CRISPR site, called pre-CRISPR RNAs (pre-crRNAs) [10, 11]. In parallel with pre-crRNA transcription, trans-activating crRNA (tracrRNA) from upstream of the CRISPR locus is transcribed to serve two essential functions: inducing maturation of pre-crRNA of the RNase III enzyme, and activating crRNA-guided DNA cleavage [12, 13]. Then, the tracrRNA:crRNA complex loads onto CRISPR-associated nuclease 9 (Cas9), forming an active ribonucleoprotein (RNP) complex. Third, the double-stranded RNA structure directs Cas9 to introduce a double strand break (DSB) in DNA at a site complementary to the crRNA spacer sequence [13]. Together, CRISPR and Cas9 play a synergistic role in bacterial resistance to phage infection and plasmid conjugation.

**Fig. 2:**
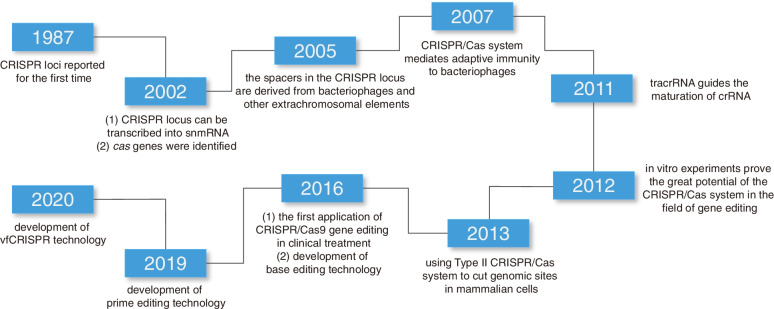
A brief history of CRISPR/Cas9 system development and associated gene editing tools. The CRISPR locus and *cas* genes were identified in 1987 and 2002 respectively. In 2005, it was discovered by RNA-sequencing that bacterial CRISPR loci contain a number of spacers derived from bacteriophage and other extrachromosomal elements. In 2007, it was confirmed that CRISPR/Cas system mediates the adaptive immunity of prokaryotes to bacteriophages. In 2012, it was confirmed that the double RNA structure formed by tracrRNA and mature crRNA instructed Cas9 to cleave DNA at the target site. In 2013, Type II CRISPR/Cas achieved precise editing of endogenous genome sites in mammalian cells. In the following years, the advent of several CRISPR/Cas9-based gene editing tools has dramatically improved the precision of genome editing and widened its extent of application. In 2016, CRISPR/Cas9 gene editing tools were first applied to clinical treatments, and subsequent clinical trials provided new insights for humans to explore cancer treatments

Changing the target of CRISPR/Cas9 only requires changing the guide RNA sequence; this prompted adaptation of the CRISPR/Cas9 genome editing system as a tool to modify genetic material in various cell types and organisms (Fig. 2) [13]. The CRISPR/Cas system encompasses three major types (types I, II, and III) and 12 subtypes (Table 1) [14]. Compared with types I and III, the type II system relies on a single Cas protein to precisely target a specific DNA sequence, so it has become the most commonly used genome editing tool [13–17]. This review mainly discusses the type II system.

**Table 1:** Comparison of three types of CRISPR-Cas systems

	Type-I	Type-II	Type-III
CRISPR-cas action			
Adaptation			
Whether to depend on PAM when selecting proto-spacers	Yes	Yes	No
Expression			
Pre-crRNA conjugates	Cascade complex	Cas9 (Csn1/Csx12) tracrRNA	Cas6 Csm (subtype III-A)/ Cmr (subtype III-B)^a^
Pre-crRNA cleavage enzymes	Cas6e subunit (subtype I-E)/Cas6f subunit (subtype I-F)	Housekeeping RNase III	Cas6
Processes to mature crRNA	1. A typical 8-nucleotide repeat fragment on the 5′ end 2. A hairpin structure on the 3′ flank	Cleavage at a fixed distance within the spacers (probably catalyzed by Cas9)	1. Cas6 is responsible for the processing step 2. Trimming the 3′ end of the crRNA further (Nucleases have not yet been identified.)
Interference			
Methods of target recognition	Cascade complex guided by crRNA	Cas9 loaded with crRNA directly	The invading DNA fragment having no base pairing to the 5′ repeat fragment of the mature crRNA (resulting in interference)
Targets cleavage enzymes	Cas3	Cas9	Cas6 or Cmr/Csm complex
Targets	DNA	DNA	DNA (III-A)/ RNA (III-B)
Whether to depend on PAM to cleaving process	Yes	Yes	No
Special systems contained	*cas3* gene	‘HNH’-type system	Polymerase and RAMP modules
Distribution of the three types of CRISPR-Cas systems	More common in Archaea	Only in Bacteria	More common in Archaea

^a^The pre-crRNA is transferred to a distinct Cas complex after Cas6 processing

In addition, the tracrRNA: crRNA structure can be a single guide RNA (sgRNA), which simplifies components of the CRISPR system and allows Cas9 to target any DNA sequence by changing the sgRNA sequence [13] as long as it is adjacent to the protospacer adjacent motif (PAM). PAM is a short sequence motif (2–5 bp) adjacent to the crRNA-targeted sequence on the invading DNA [18]. Initial binding and cleavage of DNA by sgRNA-Cas9 requires PAM recognition. Without PAM, Cas9 cannot cleave a target sequence even if completely complementary to sgRNA [19]. These motifs also are recognized during the DNA interference process of types I and II CRISPR systems [20, 21], and PAM interaction triggers Cas9 activity [22]. Analysis of the crystal structure of *Streptococcus pyogenes* Cas9 (SpCas9) in complex with a sgRNA and target DNA confirmed that if the target DNA containing PAM complements the guide RNA, an RNA:DNA hybrid called the R-loop will form, and the DNA will be cleaved [23]. DNA cleavage results from action of two different domains of Cas9 nuclease—the HNH domain cleaves the DNA strand complementary to crRNA, while the RuvC-like domain cleaves the other strand [19, 24, 25].

Because CRISPR is easier to perform and more effective than other gene editing technologies, it holds promise to accelerate development of clinical trials that incorporate gene editing. The first clinical application of CRISPR/Cas9 gene editing was in 2016, when a clinical trial delivered CRISPR gene-edited immune cells to a patient with advanced lung cancer [26]. Yet although CRISPR technology shows great potential in gene editing, its safety remains a concern. However, base editors developed by fusing the CRISPR/Cas9 system with cytidine deaminase can effectively correct genomic point mutations [27], and prime editing greatly expands the scope and capabilities of genome editing based on base editing [28]. In 2020, emergence of a technology called very fast CRISPR (vfCRISPR) enabled producing DSBs at sub-micrometer and -second levels, realizing high-resolution DNA repair research in space, time, and genomic coordinates [29]. Thus CRISPR-based technologies still hold much promise for the future of clinical gene editing, especially for cancer.

## Gene editing and transcriptional modulation of CRISPR/Cas9

Breakage of both DNA strands poses a threat to genome stability. Eukaryotic cells have two main ways to repair this fatal damage: non-homologous end joining (NHEJ), and homology directed repair (HDR). NHEJ is an error-prone repair mechanism that simply connects broken ends together, usually resulting in random insertions or deletions (indels). This may lead to frameshift mutations and thus loss of gene function [30]. Compared with NHEJ, HDR uses homologous DNA templates to reconstruct broken DNA [31]. Therefore, it is theoretically possible to accurately modify the genome sequence by generating DSBs at specific sites in the genome and introducing a donor template into the target cell [32]. Based on this principle, the type II CRISPR/Cas system accurately cleaves endogenous genomic sites in human and mouse cells [16]. Simultaneous introduction of multiple guide RNAs can achieve multiple editing of the target locus [15], proving the feasibility of CRISPR/Cas9 as a tool for eukaryotic genome editing.

A pivotal feature of Cas9 is that it can bind to specific DNA sites via guide RNA and PAM. Thus, a kind of catalytic death Cas9 (dCas9) lacking endonuclease activity can be fused with transcription activator and repressor to regulate gene expression in the whole genome. Transcription inhibition based on dCas9 is called CRISPR interference (CRISPRi), and when dCas9 is co-expressed with sgRNA, it can prevent formation of the transcription initiation complex and transcription extension. CRISPRi not only can effectively inhibit expression of multiple target genes in *Escherichia coli* simultaneously, but its effect also is reversible and does not appear to extend off-target [33]. In contrast, CRISPR activation uses dCas9 fused with activating effectors to recruit transcription machinery and RNA polymerase to activate expression of target genes [34]. By changing the sgRNA sequence, the CRISPR system represents an editable DNA binding platform to recruit related proteins to the target DNA sequence, revealing the tool’s potential to precisely regulate gene expression [35].

## Next-generation CRISPR technologies

Although CRISPR/Cas9 shows bright prospects for disease treatment, its reliance on DSBs to stimulate the gene editing process may undermine its safety. DNA breaks mediated by CRISPR/Cas9 can delete thousands of base pairs and create new genotypes, some of which may have potential pathogenic consequences in mitotically active cells [36]. For instance, CRISPR/Cas9 genome engineering in human pluripotent stem cells can generate *p53* mutations, limiting potential application for cell replacement therapy [37]. Further, the high frequency of indel mutations limits the efficiency of gene editing [38], and the incidence of CRISPR-mediated HDR observed in HEK293T cells is only 38% [39].

However, single nucleotide polymorphisms (SNPs) are the leading cause of most known genetic diseases [40]. Therefore, methods to specifically change the sequence of a single base pair at a target site without introducing DSBs are needed. Here, we review the two latest such technologies: base editors and prime editors. They have the advantages of programmability and flexibility and do not require introduction of DSBs, which may overcome the basic limitations of traditional Cas9 nuclease gene editing.

### Base editing

Base editors comprise nuclease-impaired Cas9 fused with deaminase, which can introduce specific point mutations into DNA without introducing DSBs or relying on the donor DNA template and HDR [27, 41–43]. There are currently two major types of DNA base editors: cytosine base editor (CBE) [27, 44] and adenine base editor (ABE) [42]. These two base editors can mediate all four possible conversion mutations: C to T, A to G, T to C, and G to A. However, most known natural deaminases act on RNA, and the few examples that act on DNA are only effective on single-stranded DNA (ssDNA) [45]. For CBE and ABE, the catalytically damaged Cas nuclease binds to the target DNA strand, resulting in partial denaturation of the DNA strand containing the PAM to form an R-loop [22, 46], allowing the deaminase to perform an effective deamination reaction on ssDNA.

#### Structure and mechanism of CBEs

**Fig. 3:**
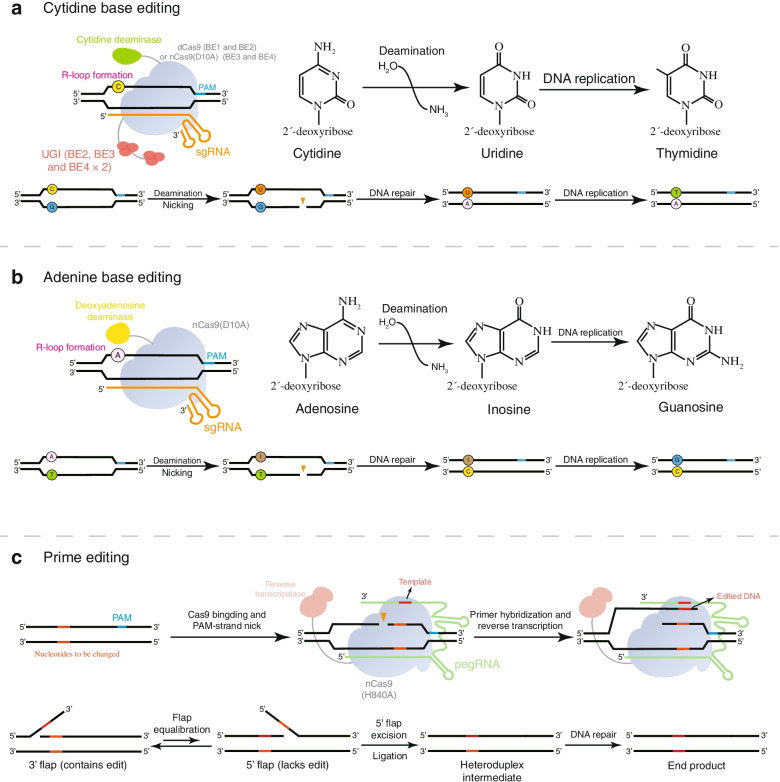
The mechanism of base editing and prime editing. **a** A cytosine base editor (CBE) uses cytidine deaminase to bind to its homologous base to catalyze the deamination reaction and convert the cytosine in the R-loop to uracil. The resulting U•G base mismatch is then converted into T•A pair after DNA replication or repair. **b** Schematic of the adenine base editor (ABE). ABE-mediated deamination converts adenosine to inosine, which is subsequently read as guanosine during DNA replication. **c** Schematic diagram of the prime editor structure and prime editing mechanism

CBE is composed of three fundamental units: cytidine deaminase, uracil DNA glycosylase (UDG) inhibitor (UGI), and a partially inactive Cas9 (nCas9) or dCas9. SgRNA guides Cas9 variants to target specific sequences to produce single strands instead of DSBs, while base deaminase catalyzes the deamination reaction to initiate base editing. In 2016, two teams designed two base editor prototypes targeting cytosine deamination. Liu and colleagues fused the rat APOBEC1 deaminase to the N-terminus of dCas9 (D10A and H840A) [27], while the Kondo team connected a PmCDA1 activation-induced cytidine deaminase ortholog from sea lamprey to the C-terminus of dCas9 [44]. CBEs use cytidine deaminase to bind to its homologous base to catalyze the deamination reaction and convert the cytosine in the R-loop into uracil, and then the mismatched U•G base pair is converted to a T•A pair through cellular DNA replication or repair mechanisms (Fig. 3a) [27].

Although the original CBE prototype (BE1) mediates effective deamination of specific cytosines in vitro, its base editing efficiency in human cells is 5–36-times lower than in vitro [27]. The dramatic decrease in efficiency may be due to the higher level of uracil excision of U•G intermediates by the base excision repair enzyme UDG, which ultimately leads to return to the original C•G base pair by the base excision repair pathway [47, 48]. UGI can effectively block the activity of human UDG [49], so introduction of UGI in BE2 can protect the U•G pair intermediate and improve base editing efficiency approximately three-fold [27]. In a further refinement, replacement of dCas9 with nCas9 produces BE3 that can create nicks in the unedited DNA strand, allowing mismatch repair (MMR) to preferentially convert guanosine to adenosine using uracil as a template [27]. Compared with previous generations of base editors, BE3 editing is more efficient and yields greater product purity, but BE3 still produces unwanted by-products at some sites [50]. Linker optimization and fusion of BE3 and a second UGI domain generated BE4, which provides significantly improved editing efficiency in mammalian cells and in vivo [50, 51].

#### Structure and mechanism of ABEs

Autogenous deamination of cytosine is the main reason for base pair conversion of C•G to T•A. Half of known pathogenic SNPs in humans are caused by spontaneous deamination of cytosine [52], but all existing base editors mediate conversion of C•G to T•A. Deamination of adenosine produces inosine, which is read as guanine by replication and transcription mechanisms [53]. Based on the above principles and research basis for CBEs, Liu and his team developed ABEs, which have a similar structure and editing mechanism to CBEs yet replace cytidine deaminase with adenosine deaminase [42]. However, the main obstacle to develop ABE is the lack of any known natural adenine deaminase that can act on ssDNA [54], and A•T to G•C editing was not initially observed in HEK293T cells [42]. Therefore, the tRNA-specific adenosine deaminase TadA from *E. coli* was evolved to generate a version (eTadA*) with activity for use as an adenosine deaminase in ABEs. ABE localizes to target DNA similarly to CBE; then, the adenosine deaminase converts adenosine to inosine to produce an I•T pair, and MMR converts the unmatched I•T pair into an I•C pair, which is further amended as a G•C pair (Fig. 3b). Unlike CBE, adding a DNA repair manipulation component such as UGI is unnecessary because intracellular inosine excision is much less efficient than that of uracil.

#### Advantages and disadvantages of base editors

Generally, mammalian cells repair DSBs mediated by CRISPR/Cas9 through two competing endogenous repair pathways: NHEJ or HDR. However, HDR in mammalian cells is inefficient, and NHEJ often leads to non-specific indels at DSB sites [38]. Base editing precisely creates specific single-nucleotide changes; it avoids DSBs caused by cleavage of the nucleic acid backbone but directly chemically modifies the target base, significantly improving product purity and reducing indels [40].

SNPs are the most common type of mutations that cause human genetic diseases [40]. In theory, base editing is particularly suitable for correcting SNPs and could correct more than 70% of disease-related SNPs. Further, base editing avoids the activation of *p53* caused by Cas9 nuclease and minimizes the adverse consequences of DSBs [55]. However, base editors must distinguish between target bases and bystander bases within a narrow window of ~ 4–10 nucleotides to achieve precise editing [56]. In addition, adenine base editing occasionally causes bystander cytosine deamination with an efficiency of 11.2% and reduces the number of suitable targets for highly specific base editing [57]. Like traditional genome editing, another potential problem with base editing is off-target effects. For clinical application of base editing, off-target effects may be a major factor in promoting tumorigenesis [58]. To address these limitations, base editor variants can minimize off-target effects while maintaining high on-target activity [59–61].

### Prime editing

Although base editors are effective at achieving the four transition mutations, improving the efficiency of correcting point mutations, and enabling application of gene editing to treat human genetic diseases, they cannot achieve eight transversion mutations and precise insertion or deletion of target gene segments [43]. In addition, DNA base editors can induce single-nucleotide variants and act nonspecifically on RNA, resulting in reduced gene editing specificity [62, 63]. In 2019, Liu and colleagues reported prime editing, which mediates directed insertion, deletion, and all 12 possible base-to-base conversions in human cells without introducing DSBs or donor DNA templates, thus greatly expanding the capabilities and applications of genome editing [28].

Prime editors consist of an nCas9 (inactivated HNH nuclease) connected to a reverse transcriptase and modified sgRNA, called prime editing guide RNA (pegRNA). pegRNA not only binds specific DNA sequences but also contains new genetic information as a template to synthesize new DNA strands. Under the guidance of pegRNA, the prime editor first binds to a specific target DNA sequence, and the Cas9 RuvC nuclease domain nicks the DNA strand containing PAM. To transfer the edited sequence from the pegRNA to target DNA, the reverse transcriptase reads the RNA and attaches corresponding bases to the end of nicked DNA, and then DNA repair machinery stably introduces the new strand into the target site (Fig. 3c). Prime editing can theoretically correct most genetic mutations associated with human genetic diseases, laying the foundation for genome editing within clinical treatment [28].

#### Advantages and disadvantages of prime editors

Prime editors offer many unique advantages. First, prime editing has no restricted editing window and can mediate all possible base transitions and transversions, providing a way to introduce new DNA sequences into specific genomic sites while avoiding DSBs. Second, the approach is highly specific with little or no off-target effects. Finally, compared to nuclease-mediated HDR, the edited result has fewer indels [28, 64, 65]. Although prime editing was successfully applied to four human cell lines and primary post-mitotic mouse cortical neurons, editing efficiency varied due to unknown factors. Understanding the factors that affect the efficiency of prime editing will help further improve the ability and scope of prime editors.

## Target discovery by CRISPR/Cas9 screens

As a high-throughput genetic screening tool, CRISPR/Cas9 has been used to analyze cancer-related gene functions as well as biological pathways [66]. Cas9 nuclease-mediated loss-of-function mutations are achieved by introducing a DSB to a constitutively spliced coding exon through specific sgRNAs. Incomplete repair of NHEJ often leads to DSB site indels, effectively mutating the sgRNA target site and resulting in gene inactivation [32]. Loss-of-function screening in cells is generally performed in two forms: arrayed or pooled.

**Fig. 4:**
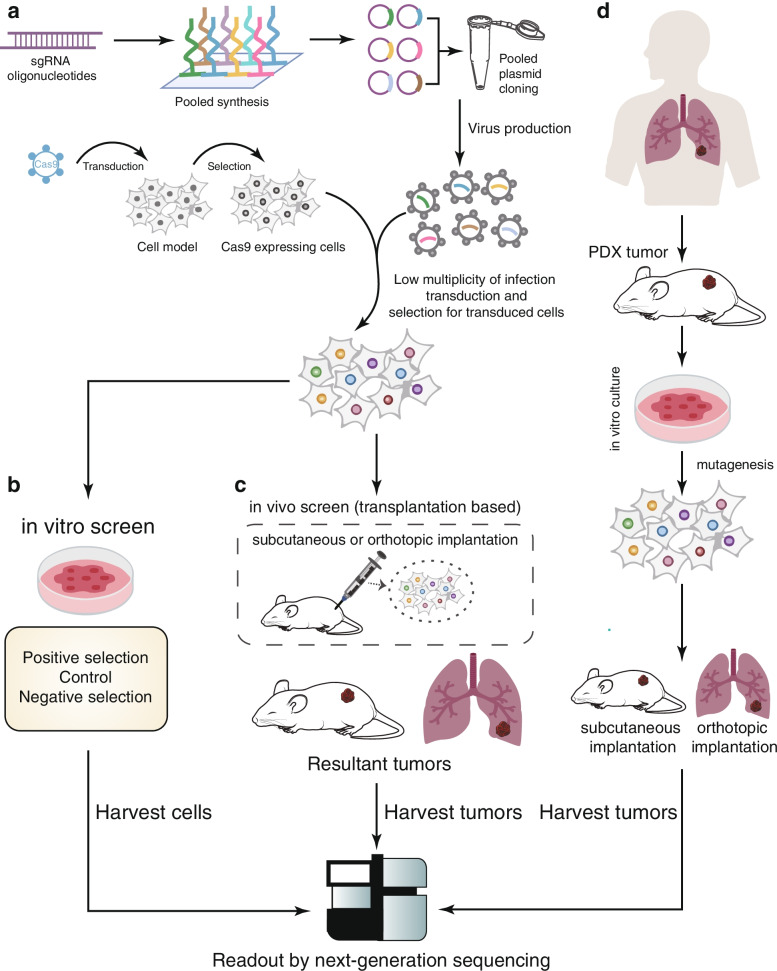
Schematic diagram of in vitro or in vivo CRISPR screening. **a** CRISPR screening begins by synthesizing oligonucleotide pools containing single guide RNA sequences and cloning them into lentiviral vectors. Lentiviruses then infect cells expressing Cas9 at low multiplicity of infection. After selection, the pool contains cells with different gene knockouts, which can be subsequently used in various screening methods. **b** In vitro screening is performed by culturing tumor cells under selective pressure such as drug treatment. **c** In vivo screening transplants the transfected cell population into immunodeficient mice in situ or subcutaneously. **d** Patient-derived xenotransplantation (PDX) is achieved by transplanting the patient’s tumor into immunodeficient mice. The PDX tumor is harvested, cultured in vitro, and genetically modified to evaluate tumor growth and response to treatment

With development of oligonucleotide library synthesis technologies, advantages such as low cost and less-intensive labor have widened use of pooled screening. CRISPR/Cas9 pooled screening requires generation of cell populations with diverse gene knockouts, which involves bioinformatics and several experimental steps (Fig. 4a). First, the sgRNA library is synthesized into a highly diverse pool of oligonucleotides, which are then cloned into the backbone of the lentiviral plasmid to produce viral particles [67]. Unlike array screening, virus particles infect Cas9-expressing cells at a low multiplicity of infection during pooled screening, so each cell may carry different sgRNA cassettes and specific gene knockouts. Subsequently, these gene-specific knockout cells are exposed to select perturbations, and then their genomic DNA is extracted. The integrated sgRNA cassette is amplified and sequenced to determine the abundance of cells with specific genes knocked-out to monitor their phenotypic effects (Fig. 4b). Use of genome-scale sgRNA libraries for gene knockout screening in human or mouse cells [68–71] demonstrates the prospect of the CRISPR/Cas9 system as an efficient loss-of-function screening method and providing a new research method for immuno-oncology. Here, we outline the latest developments in CRISPR-based immuno-oncology target screens.

### CRISPR screening in cancer cells

One of the primary purposes of CRISPR/Cas9 screening in oncology is to identify genotype-specific vulnerabilities. Targeted deletion of these genes can decrease the viability of cancer cells, providing a strategy to discover potential therapeutic targets [72]. While another application is identifying genes that work synergistically with a drug or develop resistance to the drug, combining CRISPR screening with drug perturbation can provide understanding of the mechanism of cancer response to drug treatment [69]. Receptor tyrosine kinase (RTK)/Ras/mitogen-activated protein kinase (MAPK) pathway inhibitors are clinically used to treat lung cancer and other cancers, but most patients still respond poorly to treatment. CRISPR/Cas9 gene deletion screening in lung cancer cells revealed that *KEAP1* deletion in the presence of multiple targeted RTK/Ras/MAPK pathway inhibitors changes cell metabolism, allowing cells to proliferate without MAPK signaling [73]. Thus, loss-of-function screening can help evaluate the efficacy of related drugs in clinical trials and guide treatment selection.

In carcinogenesis, neoantigens produced by somatic mutations can stimulate a potent T cell response, but mutations also can cause resistance to immunotherapy. To explore the mechanisms by which cancer cells resist immune cell killing, cancer cells transduced with sgRNA libraries were incubated with immune cells and followed by next-generation sequencing to identify sgRNAs enriched or depleted in surviving cancer cells to identify genetic perturbations that mediate resistance or sensitivity of cancer cells to immune cell killing. In a co-culture system of human CD8^+^ T cells and melanoma cells, deletion of key major histocompatibility complex (MHC)-I genes promotes cancer cell evasion from T cell killing. These key genes include *HLA-A*, *B2M*, *TAP1*, *TAP2,* and *TAPBP,* which also function in biological pathways such as interferon γ (IFN-γ) signaling, EIF2 signaling, endoplasmic reticulum stress, and protein ubiquitination [74]. Interferon signaling antagonizes cancer cells and immune cells to establish a regulatory relationship that limits innate and adaptive immune killing, and disturbance of this relationship directly affects the efficacy of immune checkpoint blockade (ICB) [75].

Natural killer (NK) cells are critical in initiating the anti-tumor response. Therefore, identifying specific genes that cause tumor cells to be sensitive or resistant to killing by NK cells may provide new targets for enhancing the anti-tumor immune response of NK cells [76]. Expression of genes related to antigen presentation (*TAP1*, *TAP2*, and *B2M*) or IFN-γ signaling (*JAK1*, *JAK2*, and *IFNGR2*) can protect tumor cells from NK cells, while *JAK1*-deficient melanoma cells regulate expression of MHC-I by attenuating the IFN-γ-driven transcription events of NK cells, sensitizing the cells to NK cell-mediated killing. Further, tumor cells resistant to T cell killing are highly sensitive to NK cell killing by enriching MHC-I-deficient clones [77]. Therefore, NK cell-based immunotherapy may be a strategy to combat tumor immune escape. Tumor cells sensitive to NK cell-induced cytotoxicity are more likely to express mesenchymal-like transcription patterns, high levels of genes controlling chromatin remodeling, and low levels of HLA-E and antigen-presenting genes. Analysis of tumor samples from patients with or without ICB treatment showed that the transcriptome related to NK cell sensitivity is significantly enriched in tumor samples of ICB non-responders [78]. Thus, some patients are more likely to benefit from NK cell-based therapy.

### CRISPR screening combined with single-cell RNA-sequencing

Despite its strengths, CRISPR-based genetic screening has inherent limitations. Pooled screening is limited to simple readouts such as cell proliferation and sortable marker proteins. Arrayed screens allow for comprehensive molecular characterization such as transcriptome profiling, yet the throughput is much lower. Instead, pooled CRISPR screening combined with single-cell RNA-sequencing directly links sgRNA expression with the transcriptome response in thousands of single cells, enabling CRISPR screening with single-cell transcriptome resolution. This method is helpful for high-throughput functional analysis of complex regulatory mechanisms and heterogeneous cell populations and provides a new method to analyze complex signaling pathways and other biological mechanisms [79].

The epithelial–mesenchymal transition (EMT) changes expression of adhesion molecules on the cell surface, enabling malignant tumor cells derived from epithelial cells to gain migration and invasion capabilities and achieve distant metastasis [80]. Figueroa et al. used a combination of single-cell trajectory analysis and high-throughput loss-of-function screening to identify receptors and transcription factors required for EMT [81]. This method is effective to identify upstream signals of cell phenotype regulatory pathways and can elucidate the genetic structure of biological processes in development and disease. For example, there is currently no targeted therapy for triple-negative breast cancer (TNBC), a highly malignant and heterogeneous tumor. BET bromodomain inhibitors (BBDIs) are a potential drug to treat TNBC, but inherent and acquired resistance of tumors to BBDIs limits their clinical application [82]. Shu et al. identified a synthetic lethal interaction with BBDIs and genes that confer resistance to BBDIs when deleted. The results showed that CDK4/6 inhibitors and paclitaxel have the strongest synergies with BBDIs, while the absence of SNF/SWI complex components leads to BBDI resistance. Subsequently, single-cell RNA-sequencing in BBDI-sensitive and -resistant cell lines showed a high degree of heterogeneity among samples, indicating that BBDI resistance can be pre-existing or acquired [83]. One advantage of the combined application of multi-omics maps and functional screening is a high degree of confidence when multiple unbiased methods are used to identify the same genes and pathways. The emergence of single-cell multi-omics technology may provide comprehensive characterization of large CRISPR libraries, forming a powerful method for large-scale analysis of cell regulation.

### CRISPR screening in T cells

T cell differentiation and functional regulation are essential for organisms to develop immunity against cancer. CRISPR/Cas9-mediated loss-of-function screening enabled high-throughput identification of critical molecules that regulate the biological behavior of human T cell lines. Due to their low transfection and transduction efficiency, delivery of Cas9 to primary immune cells is often limited [84]. However, transient delivery of Cas9 to primary T cells by electroporation overcomes this difficulty and enables gene editing in primary cells [85].

Understanding the molecular mechanisms of T cell activation may help develop effective cancer therapies. Genome-wide CRISPR screening has revealed a novel regulatory factor, FAM49B (family with sequence similarity 49 member B), that negatively regulates T cell activation. FAM49B directly interacts with the active form of the small molecule GTPase Rac. Formation of the FAM49B–Rac1 complex inhibits Rac1 activity and PAK phosphorylation, thereby affecting actin assembly. FAM49B thus inhibits T cell activation by inhibiting Rac activity and regulating cytoskeletal remodeling [86], highlighting a potential target for therapeutic development.

sgRNA lentiviral infection with Cas9 electroporation is a method that can further improve transfection efficiency, which is significant for determining T cell receptor signaling components that negatively regulate proliferation. Introduction of sgRNA cassettes by lentivirus, followed by electroporation with Cas9 protein, enables efficient and specific target gene disruption in T cells [87]. Compared with non-targeted control cells, the proliferation index of T cells lacking negative proliferation regulators such as *SOCS1*, *CBLB*, and *CD5* increases significantly, while the absence of positive TCR signaling regulator *LCP2* decreases the proliferation index [87]. Further, in vitro experiments with A375 cells show that *LCP2* knockout weakens the killing effect of T cells. In contrast, absence of negative regulatory factors including TCEB2, SOCS1, CBLB, and RASA2 significantly enhances T cell killing [87]. Further application of CRISPR screening to T cells will help explore unknown genetic circuits in primary human cells and guide development of genetically engineered T cell therapies.

Human regulatory T (T_reg_) cells are a highly specialized subset of CD4^+^ T cells essential for maintaining self-tolerance and immune homeostasis. By integrating CRISPR screening and single-cell RNA-sequencing, transcriptional regulators and downstream gene networks that may be targeted for immunotherapy have been discovered in human T_reg_ cells. For example, while HIVEP2 is not directly involved in T_reg_ cell function, HIVEP2 and SATB1 co-regulate another gene network that plays a vital role in T_reg_ cell-mediated immune suppression [88]. Discovery of the gene network of T_reg_ cells will help guide development of drug targets and design of T_reg_ cell-based therapies to relieve the immunosuppressive state in the tumor microenvironment (TME).

In summary, application of CRISPR screening in human T cells can reveal the gene regulatory network in primary cells. In the future, CRISPR screening in primary B cells and dendritic cells may provide further understanding of complex immune regulatory networks in the TME.

### In vivo CRISPR-mediated screening based on cell transplantation

Most of the above screening methods use in vitro cell models. However, in addition to target cells and effector cells, TME also contains components such as immune modulate cells and extracellular matrix, all of which can affect immune cell-mediated cancer cell killing. Therefore, in vitro models do not capture the full characteristics of the TME. In contrast, mouse models have unique advantages in assessing tumor progression and response to treatment.

Heterotopic transplantation of tumor cells is the simplest strategy to assess the tumorigenic potential of target cell lines in vivo. Cells are usually implanted under the skin of immunodeficient mice to assess tumor proliferation [89] and response to treatment (Fig. 4c) [90]. Compared with cell culture models, heterotopic tumor transplantation simulates tumor proliferation relatively accurately, thereby promoting the discovery of new metabolic-related targets [91]. However, heterotopic transplantation is generally established by subcutaneous injection of tumor cells, hence the heterotopic transplantation model cannot well characterize the TME and reveal complex immune cell regulatory networks.

To study the occurrence of tumors more realistically, an orthotopic cell implantation model or genetically engineered mouse model (GEMM) is usually used [92]. Orthotopically implanted tumors grow under the influence of TME, and are therefore considered superior to subcutaneous implanted models in studying angiogenesis and immune cell interactions (Fig. 4c) [93]. Qin et al. used genome-wide CRISPR/Cas9 gene knockout to screen candidate genes that regulate peritoneal spread of ovarian cancer (OC) in a mouse orthotopic model. Among them, *HTR1E*—a member of the 5-HT receptor family that is expressed in the ovary, endometrium, and brain—has significantly reduced expression in peritoneal disseminated OC cells, which is related to poor clinical prognosis [94]. In addition, orthotopic implantation is more suitable for survival analysis because tumor burden and associated symptoms directly lead to the death of tumor-bearing mice. However, the timing of animal sacrifice in heterotopic transplantation models is usually determined by humane endpoints defined by local IACUC guidelines. Therefore, survival data obtained from heterotopic transplant models may not accurately reflect tumor burden and associated complications [95].

GEMMs can be a platform for preclinical trials of new therapies, bridging the gap between basic medicine and clinical research, while functionally verify tumor evolution pathways and confirm genetic changes related to tumor metastasis and drug resistance. Another advantage of GEMMs is the ability to form tumors in an immunologically intact in situ environment. The signaling network formed by the TME and tumor cells regulates tumor proliferation, invasion and drug resistance [92]. In vivo CRISPR screening using GEMM has confirmed that loss of ADAR1 overcomes the resistance to immune checkpoint blockade caused by inactivation of tumor cell antigen presentation [96]. In conclusion, use of GEMMs can induce tumorigenesis in the endogenous tissue microenvironment, restore interaction of multiple TME components, and provide new insights for cancer research.

Xenotransplantation formed by ectopic or orthotopic injection of tumor cell lines in immunodeficient mice is the most frequently used platform for preclinical development of drugs. Although these cell lines are easy to obtain and use, however, they have insurmountable limitations in preclinical drug development [97]. There is evidence that the process of generating cancer cell lines can lead to irreversible changes in their biological characteristics, and since cell lines are usually established from more aggressive tumors, they do not represent complex tumor heterogeneity [98]. As an alternative, patient-derived xenograft (PDX) models involve implanting patient tumor cells or tissues into immunodeficient mice (Fig. 4d). Recent development of PDX models, especially patient-derived orthotopic xenograft (PDOX) models, established their importance in cancer biology research, drug target screening, and individualized treatment strategy development. PDX models protect the tissue structure and essential stromal elements of the primary tumor without losing its phenotype [99].

Combining CRISPR screening with PDX models provides a robust approach to identify novel therapeutic pathways for difficult-to-treat cancers. For example, the five-year survival rate of patients with pancreatic ductal adenocarcinoma is one of the lowest of all cancer types [100]. In vivo CRISPR screening in a PDOX model revealed that protein arginine methyltransferase gene 5 (*PRMT5*) is a potential drug target. At the molecular level, inhibiting PRMT5 can lead to replication protein A consumption and impaired HDR activity. Due to the accumulation of DNA damage, loss of PRMT5 activity synergistically enhances gemcitabine cytotoxicity. Combined use of gemcitabine and PRMT5 inhibitors produces in vivo conditional lethality and synergistic reduction of pancreatic ductal adenocarcinoma [101], revealing a potential novel therapeutic pathway.

As another example, most previous screening methods for acute myeloblastic leukemia (AML) therapeutic targets used in vitro models. However, this approach ignores the influence of the TME and other factors, so the transform rate of scientific research achievements is usually low. In contrast, CRISPR screening combined with a PDX model identified both previously reported AML targets and two novel genes related to AML cell survival, *SLC5A3* and *MARCH5*. Promisingly, knock-out of *SLC5A3* and *MARCH5* significantly inhibits proliferation of AML cells. Further studies have found that MARCH5 inhibition enhances the efficacy of BCL2 inhibitors such as venetoclax, further highlights the potential clinical application of targeting MARCH5 in AML [102].

Lenvatinib is an oral multikinase inhibitor and currently the first-line drug used to treat liver cancer. The global multi-center phase III REFLECT study shows that although the objective response rate of lenvatinib increased from 9.2 to 24.1% compared with sorafenib, nearly 80% of liver cancer patients still had no response to lenvatinib treatment [103]. Therefore, improving the efficacy of lenvatinib is a top priority. CRISPR/Cas9 gene screening centered on the kinome has shown that inhibition of epidermal growth factor receptor (EGFR) is synthetic lethal with lenvatinib in liver cancer. The combination of EGFR inhibitors gefitinib and lenvatinib shows effective anti-proliferation effects in EGFR-expressing liver cancer cell lines, mouse liver cancer xenograft tumor models, genetically engineered mouse orthotopic liver cancer models, and PDX liver cancer models. Further, the combination of lenvatinib and gefitinib (trial identifier NCT04642547) produced a significant clinical response in 12 patients with advanced hepatocellular carcinoma who did not respond to lenvatinib treatment. Among them, four patients achieved partial remission, and four patients with rapid progression had stable disease [104]. These results are encouraging and provide guidance to explore targeted immunotherapy combined models for liver cancer.

However, application of PDX models still has many limitations, such as replacement of the components in human TME by mouse sources and the lack of a complete immune system. Genetically humanized immunodeficient mice can simulate a more realistic human immune system, which is expected to further reveal interactions in the TME. In general, the combination of unbiased genetic screening and clinically relevant models is a practical method to explore drug resistance mechanisms of cancer cells and determine the combination of synthetic lethal drugs for cancer treatment.

## CRISPR/Cas9 delivery platforms

To fully exploit the gene editing potential of CRISPR/Cas9, they must be efficiently introduced into target cells or tissues using appropriate vectors [105]. This section will review the merits and defects of each delivery method.

### Viral vectors

Recombinant viral vectors have been developed using ability of viruses to transfer foreign genetic material into cells to deliver therapeutic genes to diseased tissues (Table 2) [106]. Among many viral vectors, adeno-associated virus (AAV), lentivirus, and adenovirus play a crucial role in genome editing therapy and have been widely used in preclinical models and clinical trials. Although modified viral vectors do not cause severe human disease, they can induce immune system-mediated clearance, which may reduce delivery efficiency [107]. Another feature of viral vectors is the ability to integrate DNA into the host genome to achieve stable gene expression, which may lead to off-target effects and insert mutation [108]. Therefore, the application of virus delivery methods is sophisticated.

**Table 2:** Viral vectors for delivery of CRISPR/Cas9 system

Delivery vehicle	Packaging capacity	Advantages	Disadvantages
Adenovirus	Approximately 8-10 kb	Efficient delivery Large cargo size	Inflammatory response
Adeno-associated virus (AAV)	Approximately 4.7 kb	Multiple serotypes Low immunogenicity Can transduce dividing and non-dividing cells in different tissues	Pre-existing neutralizing antibodies Long-term expression of Cas9 causing off-target effects
Lentivirus	Approximately 10 kb	High transduction efficiency Large cargo size Low immunogenicity Can transduce dividing and non-dividing cells in different tissues	Non-specific DNA integration causing cancer risk Complex packaging structure

#### Adenovirus

Adenovirus is a double-stranded DNA virus with a diameter of 80–100 nm. Its genome is ~ 34–43 kb in length and can package ~ 8 kb of exogenous DNA [109]. Due to its excellent ability to carry large genetic cargo, delivery efficiency of the adenovirus vector-mediated CRISPR/Cas9 module can be improved by conferring additional nuclear localization signals [110]. Continuous advancement of technology has generated adenoviral vectors lacking the viral genome, allowing loading of target DNA up to 37 kb [111]. Adenovirus can infect dividing and non-dividing cells, but one of its significant advantages is that its genome is not integrated into the host cell, reducing off-target effects and insertion mutations [112]. Nevertheless, due to its pathogenicity, introduction of adenovirus vectors can trigger the body’s immune response [113]. Although this response may enhance the killing effect on tumor cells, the neutralizing antibody response caused by activation of B cells is not conducive to subsequent vector delivery [114]. Therefore, reducing the host immune response to the adenoviral vector will greatly improve safety and delivery efficiency of this vector. Using poly(lactic/glycolic acid) copolymer to encapsulate recombinant adenovirus vectors reduces the immunogenicity of adenoviruses and enables in vitro infection in the presence of neutralizing antibodies, providing new insights for development of improved viral vectors [115].

GEMMs of human cancer are important tools to analyze the molecular mechanisms of tumorigenesis [116]. Introducing CRISPR/Cas9 into somatic cells of adult animals using adenovirus vectors induces specific chromosomal rearrangements to generate a mouse model of Eml4-Alk-driven lung cancer [117]. This strategy expands how scientists simulate human cancer in model organisms by simplifying complex and time-consuming genetic manipulations. Similarly, adenoviral vectors have been used to mediate gene editing targeting *Pten* in a mouse model of nonalcoholic steatohepatitis (NASH), in which mice injected with adenoviral vector show signs of hepatomegaly and NASH after 4 months. Even in the presence of typical adenoviral vector-related immunotoxicity in the liver, adenoviral vectors can still mediate effective *Pten* gene editing, providing a novel method to mimic human liver disease in mice [118]. GEMMs generated by site-specific recombinase technology are costly and time-consuming, but adenoviral vector-mediated CRISPR/Cas9 gene editing can effectively produce multiple subtypes of soft tissue sarcoma in wild-type mice and GEMMs. Whole-exome sequencing shows that sarcomas generated using CRISPR/Cas9 are similar to those generated using traditional recombinase technology, indicating the system’s potential to rapidly generate cancers with similar genotypes and phenotypes as traditional technologies [119].

#### Adeno-associated virus (AAVs)

AAVs consist of an icosahedral protein capsid with a diameter of ~ 26 nm and ssDNA genome of ~ 4.7 kb [120]. AAV vectors have many advantages, such as lack of pathogenicity, long-term gene expression, and the ability to infect dividing and non-dividing cells, so they are used extensively for in vivo delivery systems [120, 121]. In addition, AAV family is characterized by rich serotype diversity and has variable tropism, specifically targeting different organs [122].

Although AAVs are excellent gene therapy delivery vehicles, they still have weaknesses when used to deliver CRISPR/Cas9 in vivo. The optimal AAV vector size is 4.1–4.9 kb. Although AAV can package vectors larger than its genome size, packaging efficiency drops sharply [123]. For example, the size of the SpCas9 protein is ~ 4.2 kb, and recombinant AAV must also contain regulatory elements necessary for gene expression, so AAVs cannot be used to deliver many large gene sequences [120]. When using AAVs for transfection, SpCas9 and sgRNA must be encoded on different vectors [124, 125]. Another major problem of AAV is pre-existing neutralizing antibodies against AAV in patients with previous AAV infection, which greatly reduces therapeutic efficacy [126]. However, combining capsid modification and genome modification to produce an optimized AAV serotype vector can reduce affinity with neutralizing antibodies, thereby reducing host immune response and improving delivery efficiency [127]. In addition, long-term transgene expression of AAV also may be a risk, because continuous expression of Cas9 nuclease may cause significant off-target effects [128]. Therefore, there remain difficulties in mass production and application of AAVs.

Although there are still many challenges to overcome, people have begun to explore AAV-mediated CRISPR delivery. The AAV dual-vector system successfully targets a single gene or multiple genes in the mouse brain and characterizes the influences of genome modification on neurons [129], suggesting that that AAV-mediated genome editing can be applied to study brain gene function. Because different AAV serotypes have wide tissue tropism, AAV vector-mediated genome editing can also be used to generate animal models of cancer [130]. Platt et al. delivered a single AAV vector to the lungs of Cas9 knock-in mice to mediate *p53*, *Lkb1*, and *Kras*^*G12D*^ mutations, leading to adenocarcinoma. In addition, application of AAV to deliver sgRNA to Cas9 knock-in mice can be used for high-throughput mutagenesis in vivo to generate autochthonous mouse models of cancer [131].

#### Lentivirus

Lentivirus is a subcategory of the retrovirus family, and the lentivirus genome contains a single-stranded RNA of 7–12 kb [132]. Lentiviral vectors provide effective cell transduction in various cell types (including dividing and non-dividing cells) and shorten the culture time required for cell transfection. Compared with adenovirus or AAV vectors, lentivirus shows low cytotoxicity and immunogenicity and has minimal impact on transduced cells [133]. Because of their relative ease of use, lentiviruses are promising as in vivo delivery systems. Normally, lentivirus integrates its genome into the host genome, which can significantly extend the time for transgene expression. However, continuous expression of Cas9 may increase the risk of off-target effects and hinder application in high-precision genome editing [134]. As an alternative, integration-deficient lentiviral vectors generated by integrase mutation can greatly reduce the risk of insertion mutations [135].

Preclinical studies show that lentiviral delivery Cas9 and guide RNA targeting mutated *KRAS* significantly inhibits proliferation of cancer cells [136]. Further, lentiviral delivery of CRISPR/Cas9 targeting *BCR-ABL* significantly inhibits myelogenous leukemia cell growth and tumorigenesis, so therapies based on *ABL* gene editing may provide a potential strategy for imatinib-resistant chronic myeloid leukemia patients [137]. So far, lentiviruses have been approved for use by the U.S. Food and Drug Administration (FDA) and the European Medicines Agency [138].

#### Non-viral vectors

Safety issues remain a main bottleneck to wide clinical application of viral gene delivery, with shortcomings including insertional mutagenesis [139], immune response [107], and broad tropism [140]. As an alternative, non-viral vectors have been explored for cancer treatment due to their low immunogenicity, high biocompatibility, excellent deliverability, and low cost for large-scale production [141, 142]. Nanotechnology-based drug delivery systems will further broaden applications of CRISPR/Cas9 therapy and improve safety, providing a viable approach to overcome the challenges faced by viral vectors (Table 3).

**Table 3:** Nanotechnology-based delivery system for CRISPR/Cas9

Delivery system	Cargo options	Advantages	Disadvantages
Lipid nanoparticles	RNP plasmid DNA RNP complex Cas9 mRNA sgRNA Donor DNA	High biocompatibility Low immunogenicity Reduce off-target effects Can be mass produced Low cost	Degradation in vivo
Polymer nanoparticles	RNP plasmid DNA RNP complex Cas9 mRNA sgRNA Donor DNA	High biocompatibility Low immunogenicity Reduce off-target effects Can be mass produced Low cost	Toxicity Limited delivery efficiency
Golden nanoparticles	RNP plasmid DNA RNP complex Cas9 mRNA sgRNA Donor DNA	High biocompatibility Low immunogenicity Reduce off-target effects Can be mass produced Low cost	Limited delivery efficiency

#### Lipid nanoparticles (LNPs)

LNPs are amphiphilic systems composed of various hydrophobic and hydrophilic components, such as cationic or ionized lipids, neutral lipids such as phospholipids or cholesterol, and polyethylene glycol–lipids. LNPs are structurally different from liposomes because LNPs have no continuous lipid bilayer or large internal pool [143]. LNPs were developed as carriers to deliver a variety of molecules to cells, especially with unique advantages in nucleic acid delivery. Because nucleic acids are extremely unstable outside the cell and carry a large amount of anions, they cannot easily pass through the cell membrane. However, encapsulation in cationic liposomes allows easy delivery of nucleic acids into cells. Compared with traditional drug therapy, LNPs have unique advantages including preventing drug degradation, enabling targeted drug delivery, and reducing drug toxicity, which has generated high interest in LNPs for delivery of anti-cancer drugs [144]. Pre-clinical trials show that LNPs can successfully deliver siRNA or mRNA [145, 146], so LNPs seem to be a safe and effective delivery tool.

In the past few years, many preclinical studies of CRISPR/Cas9 delivery leveraged LNPs. Two main methods are used for LNP delivery of CRISPR/Cas9 components: delivery of Cas9 and sgRNA plasmid DNA or mRNA, or delivery of Cas9: sgRNA RNP complex. Cas9 mRNA and sgRNA can be efficiently loaded on LNPs and accurately transported to the liver of mice, effectively mediating mouse transthyretin (*Ttr*) gene editing [147]. Despite some progress, predicting and rationally designing LNPs for delivery to target tissues other than the liver for precise gene editing remains a problem. In 2020, Cheng et al. created a strategy called selective organ targeting (SORT) by adding supplementary components on the basis of traditional LNPs, precisely changing the profile of RNA delivery in the body and mediating tissue-specific gene editing [148]. SORT allows nanoparticles to deliver gene editing systems to specific organs, which is expected to promote further development of gene correction therapies.

#### Polymer nanoparticles

Polymer materials have long blood circulation, high drug bioavailability, excellent biocompatibility, and degradability, so they are considered a powerful delivery tool [149]. However, traditional methods of delivering sgRNA: Cas9 RNPs are inefficient and have poor stability to proteases in cells. The protein core and thin permeable polymeric shell form a new type of nanocapsule, which can be artificially designed for degradation or stability at different pH values. Capsule degradation breaks down the outer shell, allowing the core protein to enter the cell to perform biological functions. This method can efficiently deliver a variety of proteins to cells and also has low toxicity, opening up a new direction for delivery of sgRNA:Cas9 RNP and cancer treatment [150]. Further, in 2019 Chen et al. synthesized a thin glutathione cleavable covalent cross-linked polymer coating around the Cas9 RNP complex to generate a new nanocapsule. This nanocapsule effectively produces targeted gene editing in vitro without any obvious cytotoxicity. Topical administration of the nanocapsules in mice produces powerful gene editing capabilities [151].

In subsequent studies, Cas9 RNP was successfully delivered to 293 T cells and colorectal cancer cells and showed high genome editing activity. Importantly, nanocomplex targeting of mutated *KRAS* in cancer cells can effectively inhibit tumor growth and metastasis in tumor-bearing mouse models [152]. Guo et al. mediated effective knockdown of known breast cancer oncogene lipocalin 2 (*LCN2*) in human TNBC cells through polymer nanoparticle delivery of the CRISPR system. Loss of *LCN2* significantly inhibited the migration and mesenchymal phenotype of human TNBC cells and weakened their invasiveness [153]. In addition, Zhang et al. combined nanotechnology and genome engineering to disrupt cyclin-dependent kinase 5 (*Cdk5*), resulting in markedly decreased expression of PD-L1 on tumor cells, effectively inhibiting growth of mouse melanoma and TNBC lung metastasis [154]. Several studies showed that this polymer nanoparticle has good prospects and broad potential in transforming CRISPR genome editing into a new type of precision medicine for cancer treatment.

#### Gold nanoparticles (GNPs)

GNPs are another option for delivering CRISPR/Cas9. GNPs can combined with various components such as nucleic acids, lipids, or polymers; have relative biocompatibility; and can penetrate into many types of cells [155]. Placing diversified functional components including nucleic acids and glycoproteins on the particle surface can easily achieve functional diversity [156]. Further, pharmacokinetics of GNPs can be manipulated by adjusting their size, shape, charge, and surface modification [157–160]. GNPs equipped with engineered Cas9 protein and sgRNA can achieve ~ 90% intracellular delivery and ~ 30% gene editing efficiency, providing a new method for genomics research [161]. While HDR-based therapies probably cure most genetic diseases, it has been challenging to develop delivery vehicles that can induce HDR in the body. A delivery vehicle composed of GNPs conjugated to DNA and complexed with cationic endosomal disruptive polymers can deliver Cas9 RNP to primary cells and stem cells. This complex, called CRISPR-Gold, can induce HDR in mdx mouse primary myoblasts with minimal off-target effects [162].

Because the immune system is the first barrier for GNPs to enter the human body, it is meaningful to explore this interaction. Uptake of GNPs by immune system cells activates production of pro-inflammatory cytokines, indicating that GNPs have an immunostimulatory effect [163]. Like most cells, interaction of GNPs with various receptors on the surface of immune cells and various types of endocytosis depend on surface modification of GNPs [164, 165]. In addition, due to the unique biophysical properties of metal particles, charge and electrostatic field on the particles’ surface also significantly affect immune responses. Further research is needed to more completely define the mechanisms mediating the interaction of GNPs with the immune system.

## Clinical application of CRISPR cancer treatment

Traditional cancer treatment methods (e.g., surgery, radiotherapy, chemotherapy) can delay recurrence and prolong the survival of cancer patients, although tumor recurrence or drug resistance often leads to poor prognosis. In addition, lack of specificity of chemotherapy and radiotherapy may lead to harmful side effects and even death in some cases. Thus, new cancer therapies are still needed, and CRISPR/Cas9 technology offers potential revolutionary changes to cancer treatment.

Somatic gene therapy traditionally refers to introduction of new genetic material into somatic cells to treat disease by expressing therapeutic gene products. Gene therapy trials began as early as the 1980s, but these approaches have seen limited success because of problems such as gene silencing, host immune responses, and off-target effects [166]. Although most of these issues remain unresolved, several studies show that somatic gene therapy has good application prospects [167–170]. The first clinical trial published in 2014 used zinc finger nucleases (ZFNs) instead of CRISPR/Cas9. Patients were infused with chronic HIV viremia and received high-potency antiretroviral therapy with CD4^+^ T cells genetically modified with ZFN, with most patients showing reduced HIV DNA levels in blood [171]. Although treatment did not have a lasting effect and some serious adverse reactions have occurred, this trial set a precedent for gene therapy.

**Fig. 5:**
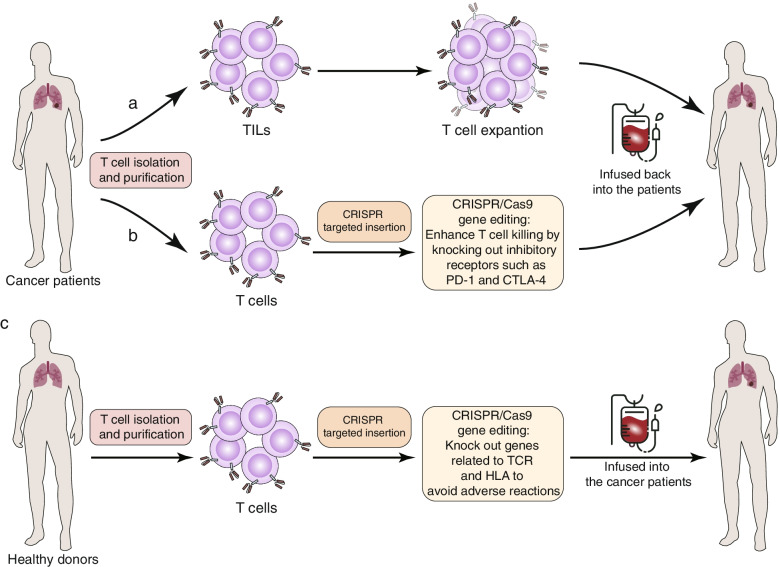
Three main approaches to adoptive cell therapy (ACT) and the application of CRISPR in them. **a** Tumor infiltrating lymphocytes (TILs) are produced by surgical removal of tumors and enrichment and amplification of TILs from tumor samples. **b** Isolation and purification of primary T cells from cancer patients, followed by CRISPR-mediated targeted insertion of chimeric antigen receptors (CAR) and engineered T cell receptors (TCR). CRISPR can then knock out immune checkpoint genes in T cells to enhance T cell function. **c** Primary T cells are isolated from healthy donors and purified, and the CRISPR system is used to introduce CAR and engineered TCR. Genes encoding endogenous TCR and human leukocyte antigen are subsequently knocked out with CRISPR/Cas9 to generate “universal” allogeneic CAR-T cells or TCR-T cells

ACT is an immunotherapy method that uses immune cells, especially T cells, to fight tumor cells. Tumor infiltrating lymphocyte therapy is one of the earliest ACTs (Fig. 5a). However, ACT is subject to many practical limitations, including difficulty isolating sufficient qualified T cells from advanced cancer patients and infants. There are two main ACT methods currently under research: chimeric antigen receptors (CAR)-T cell therapy and transgenic T cell receptor (TCR)-T cell therapy (Table 4). In most cases, autologous T cells from the patients’ peripheral blood are isolated and activated in vitro, followed by genetic engineering to express transgenic antigen receptors, including TCRs or CARs. Subsequently, modified T cells are expanded in vitro and then infused back into the patients (Fig. 5b).

**Table 4:** Registered clinical trials for the treatment of malignant tumors using CRISPR/Cas9 modified adoptive cell therapy. Data from https://clinicaltrials.gov/ (last updated 14/12/2021)

Clinical Trial Number	Phase	Target Gene	Cancer Type	Cell Type	Sponsor/Country	Recruitment Status
NCT03747965	Phase 1	*PDCD1*-KO	Adult Solid Tumor	Mesothelin-directed CAR-T cells	China	Recruiting
NCT03044743	Phase 1/2	*PDCD1*-KO	Stage IV Gastric Carcinoma Stage IV Nasopharyngeal Carcinoma T-Cell Lymphoma Stage IV Stage IV Adult Hodgkin Lymphoma Stage IV Diffuse Large B-Cell Lymphoma	EBV-CTL cells	China	Recruiting
NCT03081715	Phase 1	*PDCD1*-KO	Esophageal Cancer	Primary T-cells	China	Completed
NCT02793856	Phase 1	*PDCD1*-KO	Metastatic Non-small Cell Lung Cancer	Primary T-cells	China	Completed
NCT04417764	Phase 1	*PDCD1*-KO	Advanced Hepatocellular Carcinoma	Primary T-cells	China	Recruiting
NCT04426669	Phase 1/2	*CISH*-KO	Gastrointestinal Epithelial Cancer Gastrointestinal Neoplasms Tract Cancer Gastrointestinal Cancer Colo-rectal Cancer Pancreatic Cancer Gall Bladder Cancer Colon Cancer Esophageal Cancer Stomach Cancer	TILs	The United States	Recruiting
NCT03057912	Phase 1	*CISH*-KO	Human Papillomavirus-Related Malignant Neoplasm	TILs	China	Not yet recruiting
NCT03399448	Phase 1	*TRAC*, *TRBC*, *PDCD1*-KO	Multiple Myeloma Melanoma Synovial Sarcoma Myxoid/Round Cell Liposarcoma	NY-ESO-1 redirected autologous T cells	The United States	Completed
NCT03545815	Phase 1	*TRAC*, *TRBC*, *PDCD1*-KO	Solid Tumor	anti-mesothelin CAR-T cells	China	Recruiting
NCT03166878	Phase 1/2	*TRAC*, *TRBC*, *B2M*-KO	B Cell Leukemia B Cell Lymphoma	UCART019	China	Recruiting
NCT05037669	Phase 1	*B2M*, *CIITA*, *TRAC*-KO	Acute Lymphoblastic Leukemia Chronic Lymphocytic Leukemia Non-Hodgkin Lymphoma	CD19-specific CAR-T cells	The United States	Not yet recruiting
NCT04037566	Phase 1	*HPK1*-KO	Acute Lymphoblastic Leukemia in Relapse Acute Lymphoblastic Leukemia Refractory Lymphoma, B-Cell CD19 Positive	XYF19 CAR-T cells	China	Recruiting
NCT04557436	Phase 1	*CD52* and *TRAC*-KO	B Cell Acute Lymphoblastic Leukemia	CD19-specific CAR-T cells	The United Kingdom	Recruiting
NCT04976218	Phase 1	*TGFβR*-KO	Advanced Biliary Tract Cancer	CAR-EGFR T cells	Unknown	Not yet recruiting
NCT04767308	Early Phase 1	*CD5*-KO	CD5^+^ Relapsed/Refractory Hematopoietic Malignancies Chronic Lymphocytic Leukemia Mantle Cell Lymphoma Diffuse Large B-cell Lymphoma Follicular Lymphoma Peripheral T-cell Lymphomas	CT125A cells	Unknown	Not yet recruiting
NCT04244656	Phase 1	Unknown	Multiple Myeloma	Anti-BCMA Allogeneic CRISPR-Cas9-Engineered T Cells (CTX120)	The United States	Recruiting
NCT04438083	Phase 1	Unknown	Renal Cell Carcinoma	Allogeneic CRISPR-Cas9-Engineered T Cells (CTX130)	The United States Australia Canada Netherlands	Recruiting
NCT04502446	Phase 1	Unknown	T Cell Lymphoma	Allogeneic CRISPR-Cas9-Engineered T Cells (CTX130)	The United States Australia Canada	Recruiting
NCT04035434	Phase 1	Unknown	B-cell Malignancy Non-Hodgkin Lymphoma B-cell Lymphoma Adult B Cell Acute Lymphoblastic Leukemia	CD19-specific CAR-T cells (CTX110)	The United States Australia Canada Germany Spain	Recruiting
NCT04637763	Phase 1	Unknown	Lymphoma Non-Hodgkin Lymphoma Relapsed Non-Hodgkin Lymphoma Refractory B-Cell Non-Hodgkin Lymphoma	CD19-specific CAR-T cells (CB-010)	The United States	Recruiting
NCT03398967	Phase 1/2	Unknown	B Cell Leukemia B Cell Lymphoma	Universal Dual Specificity CD19 and CD20 or CD22 CAR-T Cells	China	Recruiting
NCT05066165	Phase 1/2	Unknown	Acute Myeloid Leukemia	WT1-directed TCR T cells	The United States The United Kingdom	Not yet recruiting
NCT04244656	Phase 1	Unknown	Multiple Myeloma	Anti-BCMA Allogeneic CRISPR-Cas9-Engineered T Cells (CTX120)	The United States Australia Canada Spain	Recruiting

TCR-T cells show great potential in immunotherapy, but their target antigen spectrum is limited and requires MHC molecules for antigen presentation. In addition, tumor cells can down-regulate expression levels of MHC molecules to achieve immune escape, which has become a major drawback of this treatment [172]. Compared with TCR-T cells, CAR-T cells recognize antigens in an MHC-independent manner, which not only eliminates human leukocyte antigen (HLA) compatibility problems between donors and recipients, but also broadens clinical application of ACT [173]. CAR-T cell therapy against CD19 has achieved striking results in treatment of relapsed and refractory acute lymphoblastic leukemia, enabling 90% of patients to achieve complete remission [174]. However, efficacy of CAR-T cells to treat solid tumors is not ideal because of the reactivity of the transduced T cells to target antigens expressed on normal tissues and rapid exhaustion of these cells in the body [175, 176]. Although CARs can recognize target antigens such as glycolipids and cell surface proteins, tumor specific antigens located in the cell and presented by MHC molecules are much more than tumor specific antigens on the cell surface, and these antigens can be recognized by TCR-T cells. Therefore, TCR-T cell therapy may be a potential method to treat solid tumors.

Although ACT has achieved promising results in tumor therapy, there remain many challenges to overcome, such as low T cell function in the immunosuppressive TME, the heterogeneity of tumor antigens, and high manufacturing cost [177]. The application of genome editing technologies in cell therapy has given rise to the next generation of cell products to improve the antitumor effect and safety of T cells and relieve immune suppression in TME. In addition, applying CRISPR/Cas9 to ACT can also improve the efficacy of immunotherapy by reducing adverse reactions and manufacturing costs, further expanding the application of immunotherapy to more cancer patients.

### CAR-T cell therapy

**Fig. 6:**
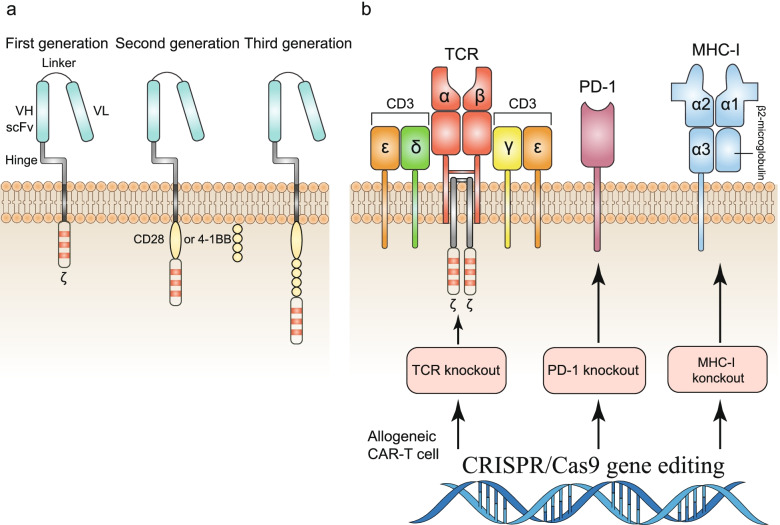
The structure of CARs and the application of CRISPR/Cas9 in CAR-T cell therapy. **a** The structure of the first to third generation CARs. **b** Knock-out of endogenous TCR sites, immune checkpoint protein and major histocompatibility complex class I molecules generates universal CAR-T cells to enhance T cell killing and avoid graft-versus-host disease

CAR is a recombinant antigen receptor which can change specificity and function of T lymphocytes, thereby generating a powerful anti-tumor response [178, 179]. CARs typically consist of single-chain variable fragments fused with a transmembrane and intracellular signaling regions, usually with one or two co-stimulatory domains (Fig. 6a) [180]. After several generations of refinement, CAR-T cells were designed to contain multiple costimulatory signals that promote continued T cell proliferation and anti-apoptosis to induce strong and sustained anti-tumor responses [181, 182]. More importantly, CAR-T cells recognize antigens in an MHC-independent manner, which allows them to recognize any HLA or tumor antigen [183]. Following the FDA approval of two autologous CAR-T cell therapies in 2017, several clinical trials showed that CAR-T cell therapy is effective for a variety of hematological and non-hematological malignancies, but there are still limitations to widespread clinical application—CRISPR genome editing may be a solution to these limitations.

Most current clinical trials use autologous CAR-T cells, which are isolated from patients, genetically edited to express the CAR structure, and then expanded in vitro and infused back into the patient [184]. This method is not only expensive [185], but the difference between the number and quality of patients’ T cells may seriously affect efficacy. In addition, the current preparation process for autologous CAR-T cell therapy is about three weeks, meaning that treatment is not immediate. In some patients with acute leukemia, such as acute promyelocytic leukemia, disease progression may occur during the process of preparing autologous CAR-T cells, and the optimal treatment opportunity may be lost [186]. Allogeneic CAR-T cells have great potential to simplify manufacturing and reduce costs (Fig. 5c) [184]. However, allogeneic CAR-T cells can recognize the recipient’s antigen through their TCR, which can cause severe graft-versus-host disease (GVHD). In addition, host-versus-graft reaction (HVGR) will quickly eliminate allogeneic T cells, thereby limiting their anti-tumor activity [183, 187]. Allogeneic αβ T cells are a key factor in mediating GVHD and HVGR, and in αβ T cells, αβ TCR heterodimers form TCR-CD3 complexes with CD3 and ζ proteins. The gene encoding the β chain contains two possible constant regions, while the gene encoding the α chain has only one [188], so disrupting the gene encoding T cell receptor constant α chain (TRAC) is the most direct and effective way to knock out the αβ TCR. In 2012, it was reported for the first time to use ZFN to gene-edit CD19-specific CAR-T cells to permanently eliminate expression of endogenous αβ TCR, thus preventing GVHD without impairing the killing ability of CAR-T cells [189]. Similarly, TALEN allows efficient multiple gene editing in human T cells. Universal CAR19 T cells generated by TALEN-mediated gene editing of *TRAC* and *CD52* produced ideal therapeutic effects in two infants with relapsed refractory CD19^+^ B cell acute lymphoblastic leukemia [190], demonstrating that gene editing technology may be a promising tool to enhance the efficacy of CAR-T therapy.

Indeed, using the CRISPR system to direct CAR to the *TRAC* locus can avoid tonic CAR signal transduction and establish effective CAR internalization and re-expression after cells are exposed to antigen, effectively delaying differentiation and exhaustion of effector T cells [191]. Although disrupting expression of CAR-T cells’ endogenous TCR does not significantly increase the killing ability of CAR-T cells, this measure does minimise the risk of autoreactivity and allogeneic reactivity. These findings reveal the immunobiological characteristics of CAR and show the direction for CRISPR/Cas9 to improve the efficacy of CAR-T cell therapy.

So far, curative effect of CAR-T cell therapy in solid tumors is not as satisfactory as in hematological malignancies, and there is still no efficacy in many solid tumor patients. This is partly due to the immunosuppressive TME and CAR-T cell exhaustion [192]. Expression of co-inhibitory molecules such as programmed death-1 (PD-1) in tumor cells and the surrounding TME results in an immunosuppressive environment [193]. Similar in principle to immune checkpoint inhibitors, PD-1-deficient CAR-T cells can be manufactured by CRISPR gene editing. PD-1 deletion enhances enhanced the antitumor activity of CAR-T cells in vitro and enhances clearance of PD-L1^+^ tumor xenografts in animal models [194]. CRISPR/Cas9-mediated endogenous T cell receptor (*TRAC*), β-2 microglobulin (*B2M*), and PD-1 (*PDCD1*) multi-gene disrupted CAR-T cells (Fig. 6b) show intensive anti-tumor activity in preclinical models of glioma, but further trials are necessary to verify the safety of these cell products to humans [195].

When T cells are exposed to continuous antigen stimulation in the tumor response, failure to eliminate antigen leads to progressive loss of effector function or dysregulation, called T cell exhaustion [196]. Similarly, CAR-T cells also acquire a depleted phenotype when entering the TME in vivo, which has become a major obstacle to CAR-T cell therapy [197, 198]. Good et al. not only summarized the characteristics of T cell exhaustion through a robust continuous antigen exposure model but also found that CAR dysfunction is related to the CD8^+^ T-to-NK-like cell transition. In addition, SOX4 and ID3 are key regulators of CAR-T cell exhaustion. CRISPR-mediated *ID3* and *SOX4* knockout can delay CAR-T cell dysfunction, laying a theoretical basis for enhancing the killing effect of CAR-T cells on solid tumors [199]. In addition to CAR-T cell dysfunction, a series of adverse reactions including allergic reactions, cytokine release syndrome and nervous system toxicity also are problems that need to be overcome in CAR-T cell therapy [200]. Future work will focus on maximizing tumor targeting of CAR-T cells while reducing toxicity.

### TCR-T cell therapy

Clinical trials have shown that CAR-T cell therapy has limited effects in solid tumors, mainly due to the lack of tumor-specific antigens, tumor heterogeneity and the suppressive immune TME. CAR can only recognize cell surface antigens, but TCR can recognize intracellular proteins, which not only expands the scope of T cells to recognize tumor antigens but also allows TCR to target cancer mutant genomes [201]. Similar to CARs, T cells can be modified with a defined TCR to make them reactive to specific tumor antigens [202]. TCR-T cell therapy shows potential against solid tumors, but identification of high-affinity specific TCRs targeting tumor-associated antigens (TAA) is a challenge for this technique, because high-affinity TAA-specific T cells are deleted by negative selection in thymus [203]. In addition, TAA expression on normal cells also may cause serious adverse reactions related to treatment. Therefore, development of TCR-T cells with minimal side effects and high proliferation and anti-tumor activity is urgently needed.

**Fig. 7:**
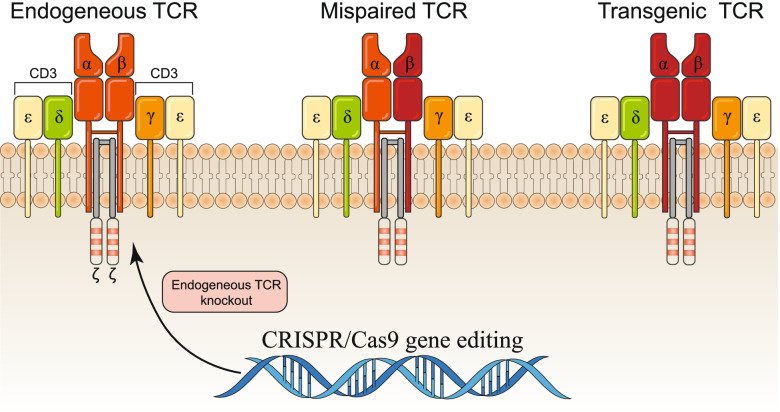
The structure of mixed TCR dimers and the application of CRISPR/Cas9 in TCR-T cell therapy. Introduction of transgenic TCRs can cause formation of new reactive TCR dimers. Knock-out of endogenous TCRs avoids the formation of mixed TCR dimers and increases the expression of transduced TCRs

One of the main problems caused by introduction of exogenous TCR into T cells is the pre-existing endogenous TCR on the recipient T cell, as introduction of exogenous TCRs can cause formation of new reactive TCR dimers (Fig. 7). These dimers, consisting of introduced TCR chains paired with the endogenous TCR α or β chains [204], are potentially pathogenic and hinder expression of transgenic TCR (tgTCR) [205]. Competition between endogenous TCR and tgTCR for CD3 molecules further limits expression of tgTCR complexes. Although introduction of additional disulfide bonds between constant domains of tgTCR can increase its expression frequency in edited T cells, there remains competition for CD3 binding [204, 206]. This series of problems may cause off-target effects, which may lead to reduced therapeutic effects and fatal autoimmunity. Using gene editing tools to mediate destruction of endogenous TCR α and β chain genes is a strategy to eliminate competition, with proven feasibility in primary T cells (Fig. 7) [207]. CRISPR/Cas9-mediated knockout of endogenous TCR-β from T cells while carrying out cancer-responsive receptor transduction significantly increases expression of tgTCR, enhancing the killing effect of engineered T cells on target cancer cells. Compared with standard TCR-transduced T cells, TCR-transduced and CRISPR-modified T cells are a thousand times more sensitive to antigens [208]. On the basis of this research, simultaneous editing of *TRAC* and T cell receptor constant β chain (*CAS*) loci produces efficient double knockout in a mass of CD8^+^ T cells, and expression level of tgTCR is further increased under dual TRAC/TRBC knockout conditions [209]. Further, elevated expression of tgTCR in the edited T cell population can enhance recognition of tumor antigens and significantly inhibit multiple myeloma growth [209]. In 2020, the first phase I human clinical trial reported using multiple CRISPR/Cas9 editing to target *TRAC*, *TRBC,* and *PDCD1* in T cells to reduce TCR mismatches and improve anti-tumor immune responses, and introduced a synthetic, cancer-specific TCR transgene (NY*-ESO-1*) to recognize tumor cells. Engineered T cells infused into the patient persisted in the body for up to 9 months, preliminarily suggesting that the combination of TCR transfer and genome editing may develop more effective and safer cancer immunotherapy [210].

CRISPR/Cas9 gene editing can specifically interfere with immune checkpoint genes, enabling TCR-T cell therapy to overcome the genes’ inhibitory effects and enhance anti-tumor immune responses, and its application in human antigen-specific cytotoxic T lymphocytes improves the anti-tumor function of PD-1-deleted cells [211]. Similarly, destroying cytotoxic T lymphocyte-associated antigen 4 molecules improves the anti-tumor efficacy of cytotoxic T lymphocytes in bladder cancer [212], providing an alternative strategy for patients who cannot tolerate immune checkpoint inhibitors. However, destruction of these suppressor genes or use of highly reactive TCR may lead to excessive T cell activation and autoimmunity. For instance, melanoma patients who received autologous peripheral lymphocytes transduced with highly reactive TCR showed persistence of genetically engineered cells in the blood and anti-tumor responses 1 month later, although patients experienced varying degrees of toxicity [213]. This demonstrates the need to consider possible toxicity of tumor-associated antigens expressed on normal tissues, and tumor-specific antigens may become targets for development. In addition, lymphocytes can be genetically edited to express costimulatory molecules, such as CD28 to eliminate the influence of inhibitory signals [179].

## Conclusion

Transforming CRISPR system into a gene editing tool has brought revolutionary changes to life sciences. Next-generation gene editing technologies have expanded versatility of CRISPR system, providing powerful new tools to study biological systems and human diseases. This article reviews two next-generation gene editing technologies: base editors and prime editors. While base editing is particularly effective in correcting common disease-causing SNPs, prime editors provide wide suitability in correcting disease-causing mutations. CRISPR technologies may become important treatments for humans to overcome genetic diseases in the near future. Base editing screening has been applied to study the relationship between gene mutation and drug resistance in cancer cells. Also, it has excellent ability to knock out specific genes without large-scale chromosomal rearrangements by prematurely introducing a premature stop codon or splice site disruption. This feature enables multiple gene knockouts in T cells to generate more efficient and safe CAR-T cells. Although prime editing is currently less used in oncology research, it provides a new strategy for developing more powerful gene mutation techniques in the future.

Development of safe and effective in vivo delivery remains the biggest challenge for widespread clinical use of CRISPR/Cas9 in human therapy. Most current clinical studies use viral vectors, but challenges such as immunogenicity, cytotoxicity and carcinogenicity still need to be conquered [214]. Rapid development of nanotechnology promotes the potential of carrier polymers and lipids. Nanocarriers can not only effectively package and protect different forms of CRISPR/Cas9 components and reduce off-target effects, but also achieve more effective blood circulation, cell uptake, and precise targeting through surface modification to reduce off-target effects. However, sustained-release system of nanocarriers still needs to overcome many physical obstacles in order to realize CRISPR/Cas editing at the tumor site and implement precise treatment [215]. Although existing nanocarriers cannot meet the requirements of large-scale clinical applications, advancement of biomaterials will help expand medical applications of genome editing in the future.

Although still in its infancy, CRISPR screening offers great promise in discovering important cancer genes and therapeutic targets. Previous studies used CRISPR screening to reveal the function of immune cells and mechanisms of interaction with cancer cells. While future studies will focus on other immune regulatory cells to uncover new regulatory pathways in the TME. PDX models and GEMMs are the best current tools to describe the histopathological characteristics of patient tumors. Therefore, it is necessary to expand research using PDX models, which will enable deep understanding of the heterogeneity among patients to inform individualized treatments.

In addition to some breakthroughs in immuno-oncology, CRISPR gene editing of human primary T cells can produce allogeneic T cells with higher antitumor activity and lower adverse reactions, which makes it possible for universal CAR-T cells to be widely used in clinical practice. Precise gene editing is expected to improve the prognosis of patients with acute leukemia, significantly reduce manufacturing costs and overcome off-target effects and GVHD associated with ACT. Further, the CRISPR/Cas9 system has great potential to target cancer-causing viruses. Using the CRISPR/Cas9 system targeting HPV18-E6 or HPV16-E6 resulted in reduced proliferation capacity and increased apoptosis in HPV-positive cervical cancer cell lines, while HPV-negative cells were not affected, providing a new idea for gene therapy in cancer [216]. In summary, the CRISPR system has had a profound impact on cancer research and will continue to play an irreplaceable role in the future.

## Data Availability

Dataset sharing is not applicable to this manuscript.

## Abbreviations

CRISPR: Clustered regularly interspaced short palindromic repeats
Cas9: CRISPR-associated nuclease 9
ACT: Adoptive T cell therapy
pre-crRNA: Pre-CRISPR RNA
tracrRNA: Trans-activating crRNA
RNP: Ribonucleoprotein
DSB: Double-strand break
sgRNA: Single guide RNA
PAM: Protospacer adjacent motif
SpCas9: *Streptococcus pyogenes* Cas9
vfCRISPR: Very fast CRISPR
NHEJ: Non-homologous end-joining
HDR: Homology directed repair
indels: Insertions or deletions
dCas9: Catalytic death Cas9
CRISPRi: CRISPR interference
SNP: Single nucleotide polymorphism
CBE: Cytosine base editor
ABE: Adenine base editor
ssDNA: Single-stranded DNA
UDG: Uracil DNA glycosylase
UGI: Uracil DNA glycosylase inhibitor
nCas9: Partially inactive Cas9
MMR: DNA mismatch repair
pegRNA: Prime editing guide RNA
RTK: Receptor tyrosine kinase
MAPK: Mitogen-activated protein kinase
MHC: Major histocompatibility complex
IFN-γ: Interferon γ
ICB: Immune checkpoint blockade
NK cell: Natural killer cell
EMT: Epithelial-mesenchymal transition
TNBC: Triple-negative breast cancer
BBDI: BET bromodomain inhibitor
FAM49B: Family with sequence similarity 49 member B
T_reg_ cell: Regulatory T cell
TME: Tumor microenvironment
GEMM: Genetically engineered mouse model
OC: Ovarian cancer
PDX: Patient-derived xenograft
PDOX: Patient-derived orthotopic xenograft
PRMT5: Protein arginine methyltransferase gene 5
AML: Acute myeloblastic leukemia
EGFR: Epidermal growth factor receptor
AAV: Adeno-associated virus
NASH: Nonalcoholic steatohepatitis
FDA: Food and Drug Administration
LNP: Lipid nanoparticle
SORT: Selective organ targeting
LCN2: Lipocalin 2
Cdk5: Cyclin-dependent kinase 5
GNP: Gold nanoparticle
ZFN: Zinc finger nuclease
CAR: Chimeric antigen receptors
TCR: T cell receptor
HLA: Human leukocyte antigen
GVHD: Graft-versus-host disease
HVGR: Host-versus-graft reaction
TRAC: T cell receptor constant α chain
PD-1: Programmed death-1
B2M: β-2 microglobulin
TAA: Tumor-associated antigen
tgTCR: Transgenic TCR
